# SMARCB1-Deficient Renal Medullary Carcinoma: A Case Report Highlighting the Value of History and Race Information

**DOI:** 10.7759/cureus.98539

**Published:** 2025-12-05

**Authors:** Habibat F Kolawole, Luisa Cardenas, Andrea Simmonds, Aly-Khan A Lalani, Michael Bonert

**Affiliations:** 1 Department of Diagnostic and Clinical Pathology, McMaster University, Hamilton, CAN; 2 Department of Oncology, The Shakir Rehmatullah Cancer Centre, Markham, CAN; 3 Department of Pathology, Trillium Health Partners, Mississauga, CAN; 4 Department of Medical Oncology, Juravinski Cancer Centre, McMaster University, Hamilton, CAN; 5 Department of Pathology and Molecular Medicine, McMaster University, Hamilton, CAN

**Keywords:** chemotherapy therapy, clinical history, renal medullary carcinoma, sickle cell trait, smarcb1/ini-1

## Abstract

SMARCB1-deficient renal medullary carcinoma (RMC) is a rare subtype of kidney cancer that predominantly affects young adults with sickle cell trait or disease. It frequently presents with metastasis at diagnosis, displays rapid disease progression, and has a generally poor prognosis. We present the case of a 25-year-old male patient with RMC who sought care for painless, gross hematuria. Imaging workup revealed a hypo-enhancing lobular mass arising from the lower pole of the right kidney. He subsequently underwent a radical right nephrectomy and a lymphadenectomy. Histopathology showed an infiltrative tumor with yolk sac-like and adenoid cystic-like morphology centered on the medulla, adjacent to benign urothelium. The lesional cells had prominent nucleoli and a moderate amount of cytoplasm. Drepanocytes were also identified. Immunohistochemistry revealed positive staining with CK7, PAX-8, and cyclin D1. SMARCB1/INI1 had a loss of staining. Pertinent negative stains included L-ALK, GATA3, CK5/6, CD10, and vimentin. Additional history was sought, and it was revealed that the patient was a person of color, had a family history of sickle cell disease, and was very physically active. The clinical history, combined with loss of SMARCB1/INI1 protein expression, led to a final diagnosis of SMARCB1-deficient RMC. Race data and relevant clinical history, including engagement in vigorous physical activity, can be diagnostically useful, as demonstrated by this case. RMC should be considered in the differential diagnosis of renal tumors in young patients. The patient was managed by the medical oncology team and was treated with chemotherapy and radiation but subsequently developed multiple metastases and passed away three years after the initial disease diagnosis.

## Introduction

SMARCB1-deficient renal medullary carcinoma (RMC) is a rare form of non-clear cell renal carcinoma with an abysmal prognosis [1]. It was described by Davis et al. in 1995 as a distinctive subtype of renal cell carcinoma that occurs almost exclusively in patients with sickle cell trait and has thus been referred to as the 'seventh sickle cell nephropathy' [2]. It was originally simply known as RMC. The 2022 World Health Organization classification of urinary and male genital tumors renamed the entity SMARCB1-deficient RMC due to the role of *SMARCB1* in the pathogenesis and diagnosis of the disease [3].

The typical patient with this disease is an adolescent or young adult (often less than 40 years old, with a median age of 28 years) who has sickle cell trait, other hemoglobinopathies, or sickle cell disease [1,4]. RMC has a male predilection, with a male-to-female ratio of 2:1, and the right kidney is preferentially involved, occurring three times as often as the left [5,6]. An increased physical activity index and significantly higher skeletal muscle (SM) surface area were found in a recent study by Shapiro et al. [6].

The presenting symptoms of RMC are often non-specific and may include abdominal or flank pain, weight loss, and fatigue. Signs of RMC may include those suggestive of urinary tract pathology, such as hematuria or a flank mass [7]. Diagnosis of RMC is more likely at an advanced or metastatic stage [8] and is generally based on histopathology. Computed tomography (CT) scans typically show a centrally localized tumor with weak and heterogeneous enhancement; it can mimic an upper tract urothelial carcinoma that invades the renal parenchyma [9].

Histopathologic features of RMC include an infiltrative tumor composed of poorly differentiated cells with variable architectural patterns, including solid, microcystic, reticular, or sarcomatoid, with mixed inflammatory cell infiltration originating from the renal medullary boundary, combined with the visualization of sickled red blood cells (RBCs). Loss of SMARCB1/INI1 protein expression on immunohistochemistry supports the diagnosis of RMC. RMC has been described as having a rapid disease progression, frequent metastases at diagnosis, and often fatal outcomes with a reported median overall survival (OS) of 13 months [5]. We present a case of RMC in a 25-year-old male patient.

## Case presentation

A previously healthy 25-year-old male presented with intermittent right-sided flank pain and painless gross hematuria. He denied nausea, vomiting, loss of appetite, weight loss, night sweats, and prior trauma to the flank. He was a lifetime non-smoker and was not on any medications. He reported being physically active and performing a significant amount of exercise. He had no family history of urological malignancy. The physical examination was unremarkable.

Laboratory investigations revealed a serum creatinine of 120 mg/dL with an eGFR of 72, an elevated alkaline phosphatase of 152 IU/L, and the presence of blood and trace leukocytes on urinalysis. Urine cytology was negative for high-grade urothelial carcinoma.

**Figure 1:**
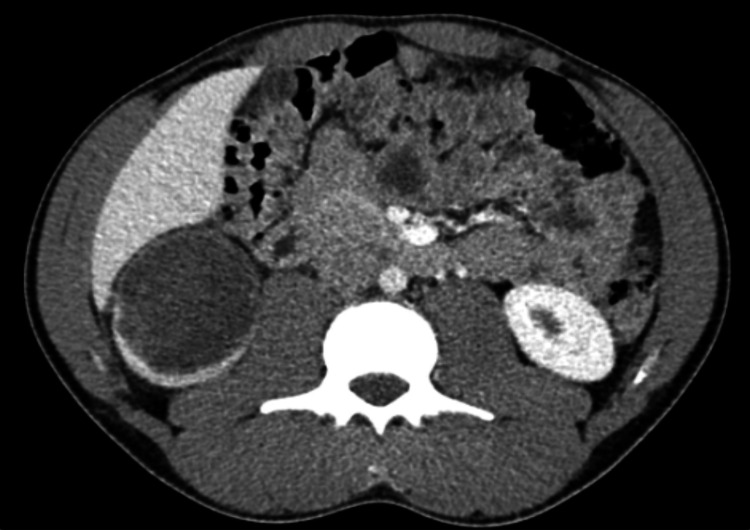
Abdomino-pelvic CT scan Cystic right lower renal cortical mass with right hypoenhancing right retroperitoneal lymph node suspicious for malignancy. Increased skeletal muscle surface area was identified in retrospect: the psoas muscles and erector spinae muscles are hypertrophic.

A contrast-enhanced computed tomography (CT) of the abdomen and pelvis showed a hypoenhancing lobular mass arising from the lower pole of the right renal cortex measuring 6.8 x 6.5 x 6.3 cm (Figure 1). The mass was mostly cystic with irregular septations and mural thickening. In addition, there was a 1.2 cm hypoenhancing right retroperitoneal node posterior to the inferior vena cava at the level of the kidney, suspicious for metastatic lymphadenopathy. In retrospect, the SM surface area appeared increased on the CT scan. There was no evidence of metastatic disease to the liver, lungs, bones, or lymph nodes on the CT scan. Based on the imaging findings, the diagnosis of malignant renal mass was made, and the patient underwent a radical nephrectomy with lymphadenectomy.

Grossly, the kidney measured 14.5 x 8.0 x 5.7 cm and had renal capsular involvement and perinephric fat invasion. The tumor measured 7.3 cm in its greatest dimension and had a solid, gray-white cut surface with infiltrating borders. An accompanying retroperitoneal lymph node measured 2.1 cm in its greatest dimension and had a pale tan cut surface, consistent with metastasis.

**Figure 2:**
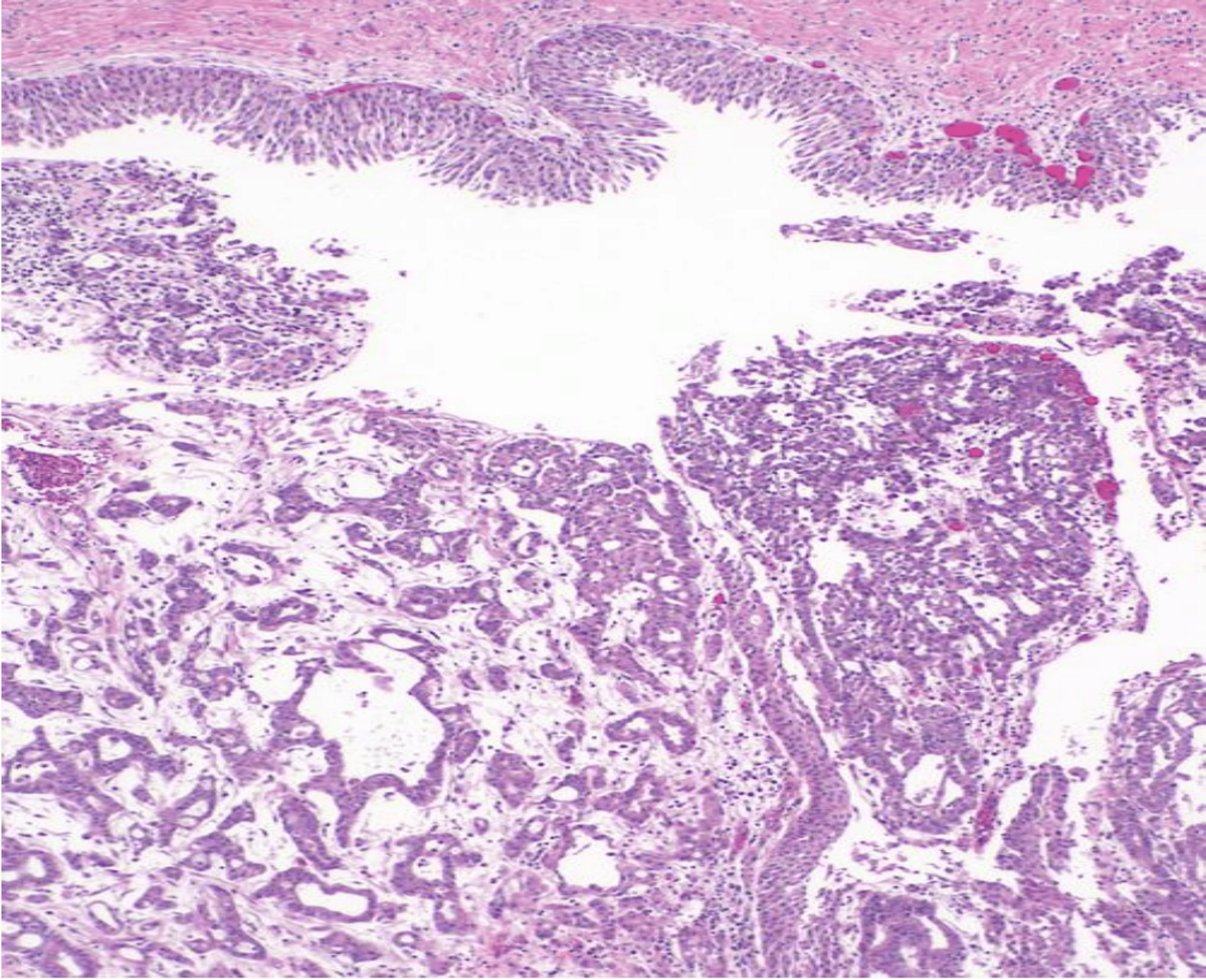
Hematoxylin and eosin (H&E) stained sections of tumor at ×100 magnification Tumor with yolk sac-like growth pattern and a benign urothelium in the background.

**Figure 3:**
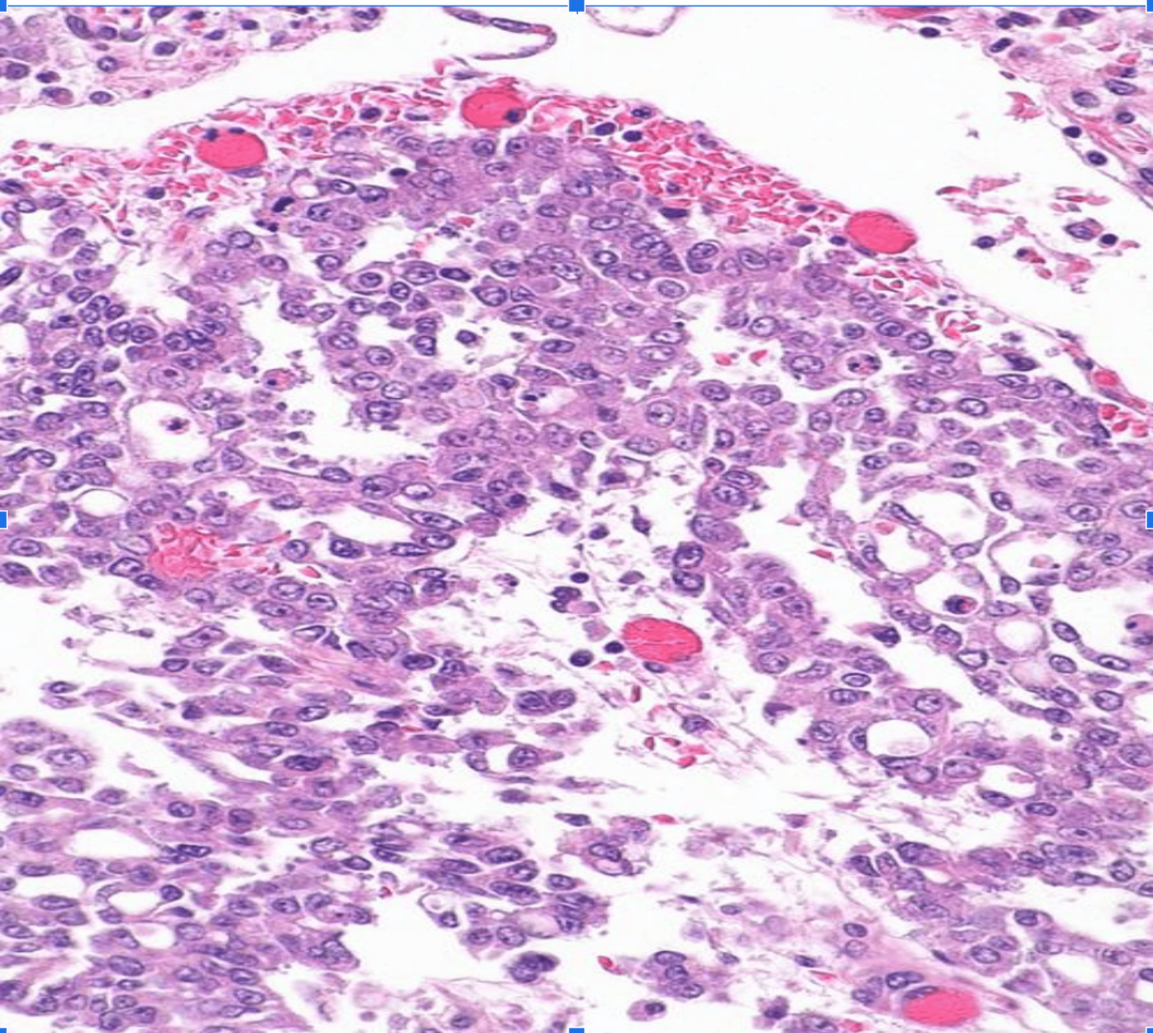
Photomicrograph of hematoxylin and eosin (H&E)-stained sections of the tumor Intermediate magnification showing the tumor.

**Figure 4:**
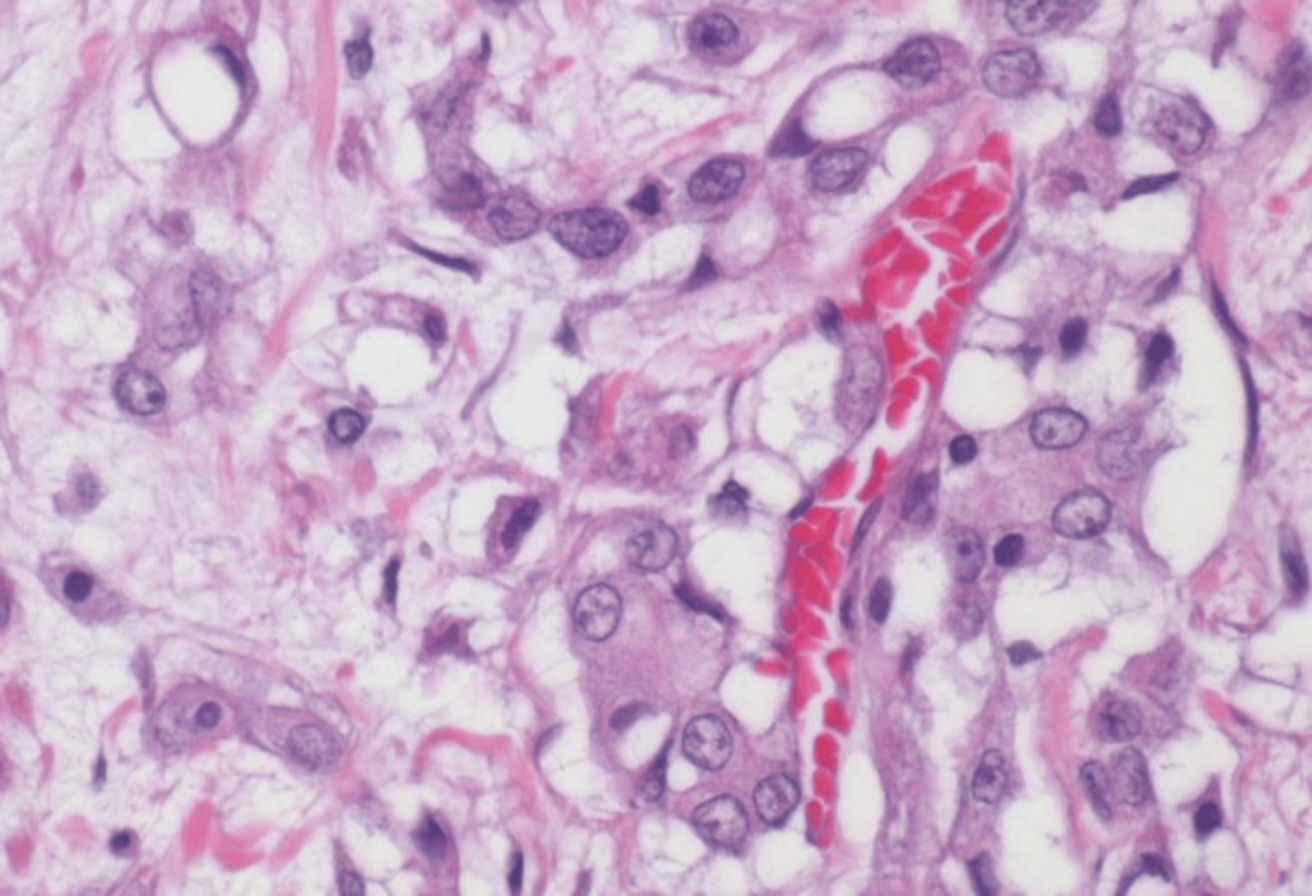
High-magnification micrograph of the tumor (H&E stain) Tumor with drepanocytes (sickle cells) in a blood vessel.

Histology showed an infiltrating, poorly differentiated epithelial neoplasm with solid, yolk sac and microcystic growth patterns. Tumor nuclei were highly pleomorphic and exhibited abnormal mitoses (Figures 2, 3). Benign urothelium was present in the background. Sickled RBCs (drepanocytes) (Figure 4) and perineural invasion were also identified. The retroperitoneal lymph node was positive for a metastatic tumor.

The clinical history on the pathology requisition was insufficient; it lacked information regarding the patient's race or family history. This led the pathologist to contact the managing physician to obtain more clinical information and ascertain whether there was a personal or family history of sickle cell disease or trait. Additional clinical history revealed the patient to be a person of color with a family history of sickle cell disease. Subsequently, hemoglobin electrophoresis confirmed the presence of the hemoglobin S trait. The time required to request and obtain additional clinical information led to a delay in the sign-out of the case.

**Figure 5:**
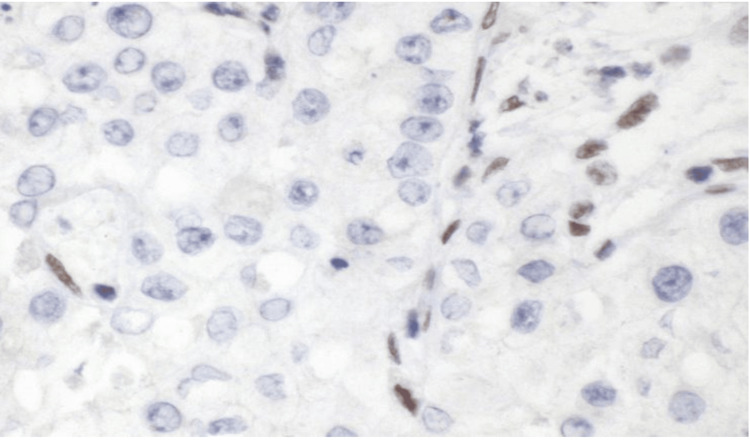
Micrograph showing loss of SMARCB1 staining in the tumor cells. Note the retained staining in the stromal cells (internal control)

Ancillary studies, including SMARCB1/INI1, showed loss of expression (Figure 5), which led to a final diagnosis of SMARCB1-deficient RMC. The tumor cells were positive for PAX8, cyclin D1, and CK7. The following stains were negative: L-ALK (D5F3), GATA3, CK5/6, MA903, CK20, CD10, and vimentin.

The patient qualified for the serum-based provincial genetic testing program, which includes a hereditary renal cancer panel. The genetic testing examined the genes *BAP1*, *FH*, *FLCN*, *MET*, *MITF*, *PTEN*, *SDHA*, *SDHAF2*, *SDHB*, *SDHC*, *SDHD*, *TP53*, *TSC1*, *TSC2*, *VHL*, and *SMARCB1*. No clinically significant sequence or copy number variants were detected in the 16 genes analyzed.

Three months after surgery, the patient developed metastatic disease in the lungs, mediastinal lymph nodes, and bones. He subsequently enrolled in the KEYNOTE-B61 trial, in which he received pembrolizumab and lenvatinib. He later developed multiple new liver metastases and lytic bone lesions for which he received radiation therapy. He subsequently received second-line carboplatin/paclitaxel, third-line doxorubicin/gemcitabine, fourth-line erlotinib/bevacizumab, and fifth-line panitumumab/Nab-paclitaxel/ and carboplatin. He subsequently developed significant disease progression with multiple metastatic lesions to the liver, bones, and lungs and passed away three years after the initial disease diagnosis.

## Discussion

RMC is an extremely rare neoplasm originating from the renal collecting duct, accounting for less than 0.5% of all renal carcinomas [1]. It occurs frequently in patients with sickle cell trait, often in the form of hemoglobin AS (HbAS). However, a few cases have been reported in individuals with sickle cell anemia (homozygous SS disease), HbS/β-thalassemia, and HbSC [1].

The prevalence of RMC may be underestimated due to underdiagnosis. Considering RMC in the differential diagnosis, attention to histologic clues, special immunostains, imaging, and a complete history are necessary to differentiate RMC from its possible mimics, including renal collecting duct carcinoma, ALK-rearranged RCC, poorly differentiated RCC, urothelial carcinoma, and extrarenal malignancies [10].

The disease frequently affects children and young adults, with most patients presenting between the ages of 11 and 39 years. Typically, patients either have a prior diagnosis or are diagnosed with sickle cell trait during workup for RMC, as seen with our case [11]. RMC has a dismal clinical outcome, and it is characterized by early and widespread metastases; thus, most cases are diagnosed in late and advanced stages with a poor prognosis [5].

It is postulated that RMC originates from the renal papillae or calyceal epithelium due to chronic renal medullary hypoxia in the context of sickled RBCs in individuals with sickle cell trait. This postulation is supported by the strong expression of vascular endothelial growth factor (VEGF) and hypoxia-inducible factor associated with these tumors [12]. High-intensity exercise, commonly seen in athletic settings and military service, has been found to aggravate renal hypoxia in the setting of sickle cell trait [6]. This finding is associated with an increased SM surface area identified on CT scans and a higher incidence of RMC in this patient population. In retrospect, our patient exhibited an increased muscle surface area on the initial abdominal CT scan.

Loss of SMARCB1/INI1 expression is a hallmark feature in RMC and is thought to play a major role in its pathogenesis [5,13]. It is recommended that RMC should be considered in the differential diagnosis for the workup of renal tumors arising in young patients less than 50 years old with sickle cell trait who present with a history of high-intensity exercise, hematuria, flank pain, weight loss, or symptoms associated with metastatic disease [6,14]. The common sites of metastasis include the regional lymph nodes, adrenal glands, lungs, liver, inferior vena cava, and peritoneum [15]. The history of sickle cell trait may be difficult to ascertain, as it is not routinely inquired about, particularly in Western countries, and among patients who have not previously been diagnosed or had complications related to the sickle cell trait.

Classic CT scan findings include a large mass in the right kidney (average size of 7 cm), associated with satellite lesions and intratumoral necrosis [11]. This is in concordance with our patient who had a 6.8 cm right renal cortex mass with a right retroperitoneal lymph node metastasis.

Grossly, RMCs appear as infiltrative solitary grayish-white masses with prominent necrosis and hemorrhage arising from the renal pelvis [4,15]. Histologic examination often reveals an infiltrative tumor composed of solid sheets of poorly differentiated epithelial cells with an inflammatory infiltrate consisting of neutrophils and lymphocytes. Various microscopic patterns, including solid, reticular, tubular, adenoid cystic, trabecular, cribriform, micropapillary, and sarcomatoid morphology, have been described [14].

The visualization of sickled RBCs (drepanocytes) and the loss of SMARCB1/INI1 expression are considered hallmarks of RMC [5]. The tumor cells frequently express cytokeratin AE1/AE3, low-molecular-weight cytokeratin, vimentin, cyclin D1, hypoxia-inducible factors (HIF), and VEGF. Variability in the expression rate of high-molecular-weight cytokeratin is common [16]. Treatment for RMC is difficult, and it has been reported to have a median OS of 13 months [5]. The standard-of-care treatment for localized, non-metastatic RMC is nephrectomy followed by close monitoring [5]. The rarity of RMC and its poor prognosis have resulted in most therapeutic choices being informed mainly by case reports and small patient series rather than randomized clinical trials. Msaouel et al. in 2019 updated the proposed clinical guidelines by Berkermann et al. in 2016 for the clinical management of patients with RMC, with the major change being that nephrectomy should be performed only if there is a response to chemotherapy within a perioperative scheme [16]. They also proposed that upfront systemic therapy should be considered for most patients with RMC, including those with localized disease. Various treatment modalities, including surgery, chemotherapy, radiotherapy, and biological agents, have been used in the management of RMC with moderate success. Our patient had a remarkable survival of three years, considering the dismal prognosis associated with RMC.

A detailed clinical history, including genotype and race information, is necessary in the diagnosis of RMC, as highlighted by this case. Collection of race data is particularly relevant, as RMC occurs almost exclusively in patients with sickle cell trait [1]. A complete clinical history that includes the patient's race, family history of sickle cell trait, or the patient's genotype would have led to a faster turnaround time. The pathology requisition is an effective tool for the provision of relevant data in the diagnostic workup. There is a need to improve the provision of complete and clinically relevant information to the laboratory, and ideally, standardized structured forms with elements relevant to the submitted specimen, similar to synoptic pathology reports, should be implemented [17].

Many individuals are unaware that they carry the sickle cell trait; hence, hemoglobin electrophoresis should be ordered in all suspected cases of RMC regardless of race. Due to the strong association between RMC and sickle cell trait, we believe that hemoglobin electrophoresis should be included in the genetic screening for hereditary renal cancers. In addition, it should be noted that patients with the sickle cell trait or sickle hemoglobinopathies may develop renal cell carcinoma other than RMC [18].

## Conclusions

SMARCB1-deficient RMC is a rare, difficult-to-treat kidney tumor with a uniformly poor prognosis that occurs in adolescents and young adults in association with sickle cell trait. Here, we report a case of RMC in a 25-year-old male. The diagnosis in this case was challenging, partly due to the absence of clinical information. Race data can be diagnostically useful, as demonstrated by this case. RMC should be considered in the differential diagnosis of renal tumors in young patients. In general, the provision of race data and relevant clinical data would be highly desirable.

A mandatory standardized history, relevant to the submitted specimen (analogous to the cancer synoptic report in pathology), could improve communication and likely speed up the diagnostic process by limiting unnecessary ancillary testing.
